# Biofeedback in Pediatric, Adolescent, and Young Adult Cancer Care: A Systematic Review

**DOI:** 10.3390/children12080998

**Published:** 2025-07-29

**Authors:** Marie Barnett, Shari A. Langer, Konstantina Matsoukas, Sanjana Dugad, Anelisa Mdleleni, Inna Khazan

**Affiliations:** 1 Memorial Sloan Kettering Cancer Center, Department of Psychiatry, New York, NY 10065, USA; matsoukk@mskcc.org (K.M.); contact@formyndstudio.com (S.D.); mdlelea@mskcc.org (A.M.); 2 Inova L.J. Murphy Children’s Hospital/Inova Peterson Life with Cancer, Fairfax, VA 22031, USA; shari.langer@inova.org; 3 Harvard Medical School, Department of Psychiatry, Boston, MA 02115, USA; inna.khazan@bostonhealthpsychology.com

**Keywords:** biofeedback, systematic review, oncology, pediatric, adolescent and young adult (AYA)

## Abstract

**Background/Objectives**: Biofeedback interventions are increasingly utilized in pediatric and adult care, with evidence in treating specific medical conditions and specific symptoms. However, evidence supporting their efficacy among children and adolescents and young adults (AYAs, aged 15–39) with cancer is limited. The aims of this systematic review are to present, assess, and synthesize the existing research on biofeedback in pediatric and AYA oncology, identify gaps in biofeedback research within this population, and provide recommendations for future research and clinical implications. **Methods**: A systematic search for articles was conducted using six bibliographic databases—PubMed/MEDLINE (NLM), EMBASE (Elsevier), CINAHL (EBSCO), SPORTDiscus (EBSCO), PsycINFO (OVID), and PEDro (NeuRA)—with an update on 5/7/2025. Included were studies involving pediatric/AYA oncology participants (0–39 years old) and those receiving at least one biofeedback modality. The methodological quality and risk of bias among included articles were assessed using the Cochrane Risk of Bias (ROB) Tool (modified version for non-randomized studies). A narrative synthesis of included studies examined the type of cancer studied, type of biofeedback used, study designs and methodological quality, and key outcomes evaluated. **Results**: While the literature suggests that biofeedback may offer beneficial outcomes for managing various pediatric/AYA oncology-related symptoms, such as pain, anxiety, and fatigue, only 8 studies out of 1013 screened (<1%) met inclusion criteria. Limitations included low study quality (small sample sizes, lack of control groups, and methodological inconsistencies). **Conclusions**: While biofeedback shows promise as a feasible and effective intervention, there is a call to action for well-designed, methodologically rigorous studies to substantiate its effectiveness and inform evidence-based practice specifically for pediatric/AYA oncology patients and clinicians.

## 1. Introduction

Children and adolescents and young adults (AYAs, defined as those aged 15–39) with cancer often exhibit remarkable resilience; nevertheless, the psychological impact of cancer and its treatment remains substantial, as these young patients face not only the physiological burden of illness and treatment, but the emotional challenges it brings. Symptoms such as anxiety, depression, pain, fatigue, and sleep disturbances are common and can persist beyond the completion of medical treatment [1,2,3]. Addressing these mental health challenges is crucial for improving both quality of life and treatment outcomes. Biofeedback, a non-invasive intervention that enables individuals to gain control over physiological functions, has shown promise in mitigating these symptoms [4,5]. While biofeedback has been extensively studied in adult populations across various medical conditions, including cancer, research in pediatric and AYA oncology remains limited [6,7]. This gap underscores the need for a systematic examination of existing studies on biofeedback interventions in this cancer population.

### 1.1. What Is Biofeedback?

Biofeedback is a technique that enables individuals to develop greater awareness and voluntary control over physiological and mental functions using real-time feedback from precise instruments. Since its emergence as a field in the 1960s, biofeedback has been defined in various ways. In 2007, a consensus definition was established by leading organizations, including the Association for Applied Psychophysiology and Biofeedback (AAPB), the Biofeedback Certification International Alliance (BCIA), and the International Society for Neurofeedback and Research (ISNR) [8]. They defined biofeedback as *“a process that enables an individual to learn how to change physiological activity for the purpose of improving health and performance. Precise instruments measure physiological activity such as brainwaves, heart function, breathing, muscle activity, and skin temperature. These instruments rapidly and accurately ‘feed back’ information to the user. The presentation of this information—often in conjunction with changes in thinking, emotions, and behavior—supports desired physiological changes. Over time, these changes can endure without continued use of an instrument.”*

### 1.2. Biofeedback Modalities

Several biofeedback modalities have been developed, each targeting different physiological systems:

- Cardiovascular Biofeedback: This modality measures heart rate (HR) and heart rate variability (HRV). HRV, the variation in time between consecutive heartbeats, is a crucial indicator of autonomic nervous system balance and overall health [9]. It has been widely studied in relation to cardiovascular health, mental well-being, stress regulation, and performance optimization. Research has demonstrated that low HRV is associated with a range of psychophysiological conditions, including anxiety, depression, post-traumatic stress disorder (PTSD), chronic pain, gastrointestinal disorders, and high blood pressure [10,11,12,13,14,15]. In adult cancer populations, HRV has been identified as a prognostic marker, with lower HRV predicting poorer treatment outcomes [16,17].
- Respiratory Biofeedback: This involves monitoring the breathing rate and patterns, as well as carbon dioxide levels, to optimize respiratory function and reduce symptoms of anxiety, asthma, and panic disorder [18,19,20].
- Neuromuscular Biofeedback: Using surface electromyography (sEMG), this modality measures muscle tension and can be used to manage pain and improve physical function [21,22,23].
- Skin Conductance Biofeedback, Also Known As Galvanic Skin Response: This method assesses eccrine sweat gland activity to provide insight into sympathetic nervous system activity, particularly in response to stress [24,25].
- Peripheral Skin Temperature Biofeedback: This modality measures temperature changes in the extremities as an indicator of stress and autonomic function [26,27].
- Neurofeedback (EEG Biofeedback): This modality monitors brainwave activity using electroencephalography (EEG) to enhance brain health and cognitive function [28,29].

### 1.3. Efficacy of Biofeedback in Adult Populations

Biofeedback has been widely studied as an effective intervention for various adult conditions, including chronic pain, anxiety, depression, PTSD, gastrointestinal disorders, epilepsy, headache disorders, and hypertension [30,31,32,33,34,35,36,37,38]. Studies have demonstrated that biofeedback can significantly reduce physiological stress responses, improve emotional regulation, enhance autonomic balance, and improve chronic disease management, particularly through HRV biofeedback [4,5,39,40]. Furthermore, biofeedback has been incorporated into multimodal treatment approaches for typically difficult-to-treat conditions such as fibromyalgia and chronic fatigue syndrome, demonstrating its broad applicability [41,42].

### 1.4. Biofeedback in Adult Oncology

Biofeedback interventions have been increasingly explored in oncology for symptom management, with studies indicating benefits in reducing pain, fatigue, anxiety, and depression among cancer patients. HRV biofeedback has been shown to improve sleep quality, autonomic regulation, symptom management, and overall well-being in cancer populations [43,44,45,46]. Studies on lung cancer patients suggest that biofeedback-assisted stress management can help mitigate psychological distress and improve coping strategies [47]. A study on breast cancer patients showed that electromyography (EMG) and HRV biofeedback reduce depression, anxiety, and stress following a mastectomy [48]. Additionally, EMG biofeedback and biofeedback combined with music training have been found to be effective in managing cancer-related pain and stress in palliative care settings, highlighting biofeedback’s potential as a complementary intervention [49,50]. Other studies have demonstrated reductions in chemotherapy- and cancer-related anxiety and improved emotional resilience following neurofeedback and skin conductance biofeedback training [51,52].

### 1.5. Biofeedback in Pediatric and AYA Populations

Although research on biofeedback in pediatric and AYA (defined by the National Comprehensive Cancer Network (NCCN) as those aged 15 to 39 [53]) populations is less extensive than in adults, available studies indicate its efficacy for managing conditions such as pediatric anxiety, migraines, ADHD, epilepsy, and chronic pain [6,28,54,55,56,57]. Systematic reviews suggest that HRV biofeedback can significantly reduce symptoms of anxiety and depression in children with chronic illnesses [58,59]. Furthermore, biofeedback has been successfully implemented to alleviate procedural distress, such as anxiety during blood draws [60]. Expanding targeted research in this area could provide valuable insights into optimizing biofeedback interventions for young cancer patients that encounter symptoms and scenarios where biofeedback has demonstrated effectiveness.

### 1.6. The Need for Research in Pediatric and AYA Oncology

Despite the demonstrated efficacy of biofeedback in adult cancer patients and pediatric populations with other medical conditions, there remains a significant gap in research on its application in pediatric and AYA oncology. Given the psychological and physiological challenges faced by this age group when undergoing treatment, biofeedback represents a promising, non-invasive intervention to enhance coping mechanisms, improve quality of life, and potentially influence treatment outcomes. In examining the paucity of research among this population, a critical question arises: what do existing studies reveal about the use, efficacy, and application of biofeedback interventions in pediatric and AYA oncology, and what gaps remain that should be addressed in future research to enhance clinical care for this unique population?

This systematic review aims to (1) present, assess, and synthesize the existing research on biofeedback specifically among pediatric and AYA oncology populations, and (2) identify gaps in biofeedback research within this population and provide recommendations for future research and clinical implications.

## 2. Materials and Methods

### 2.1. Literature Search

Prior to the initial search, this systematic review was registered with PROSPERO (ID: CRD42023454763). A systematic search for articles was then conducted using six bibliographic databases—PubMed/MEDLINE (National Library of Medicine, Washington, DC, USA), EMBASE (Elsevier, Amsterdam, The Netherlands), CINAHL (EBSCO, Ipswich, MA, USA), SPORTDiscus (EBSCO, Ipswich, MA, USA), PsycINFO (OVID, New York, NY, USA), and PEDro (Neuroscience Research Australia, Sydney, Australia)—with the final search updates conducted on 5 July 2025. The search results were limited to the English language and to Human studies. No date, publication type, or study design limits were applied. The search strategy included both keyword and controlled vocabulary terms describing three main concepts: biofeedback, cancer/childhood cancer, and pediatric/AYA [61,62]. The results of the three concept searches were combined using the Boolean operator AND (note: a simplified version of the search was adapted for the PEDro database). The Scopus (Elsevier) database was used to carry out a citation search using the included studies identified during full-text screening. Covidence systematic review software (Veritas Health Innovation, Melbourne, Australia; available at www.covidence.org) was used to identify duplicates (see Supplementary Materials for full search strategies for all databases).

### 2.2. Study Inclusion Criteria

Studies were deemed eligible for inclusion if they were in English and included any biofeedback intervention or modality that distinguished results among pediatric and adolescent or young adult oncology patients aged 0–39. Articles were excluded if no biofeedback was included, the publication language was not English, the study focused on the adult population only or did not differentiate age in results, the field of study was non-cancer/oncology, or the publication type was not a peer-reviewed article (e.g., book chapter, dissertation).

### 2.3. Study Selection and Characteristics

All titles and abstracts were independently reviewed for eligibility by two co-authors (from a pool of five possible reviewers). Any discrepancies over agreement for inclusion were discussed and resolved. At the full-text stage, reasons for exclusion were recorded by the two authors. All reviewers then met as a group and compared full-text article reviews to resolve any discrepancies and make final decisions regarding inclusion. The Scopus (Elsevier) database was used to carry out a citation search using the included studies identified during full-text screening. Database search results were captured using the Endnote citation management software program (Version EndNote 21, Clarivate Analytics, Philadelphia, PA, USA) and screened using Covidence systematic review software (Veritas Health Innovation, Melbourne, Australia; available at www.covidence.org).

The methodological quality and risk of bias among included articles were assessed by two reviewers (SD, AM) with the Cochrane Risk of Bias (ROB) Tool (modified version for non-randomized studies) [63]. The tool considers seven domains: (1) random sequence generation; (2) allocation concealment; (3) selective reporting; (4) blinding (participants/personnel); (5) blinding (outcome assessment); (6) incomplete outcome data; (7) other sources of bias. The ROB was divided into three levels: high, low, and unclear risk. Then studies were categorized into three levels of overall risk according to the number of components for which high risk potentially existed: high risk (five or more), moderate risk (three or four), and low risk (two or less).

## 3. Results

**Figure 1:**
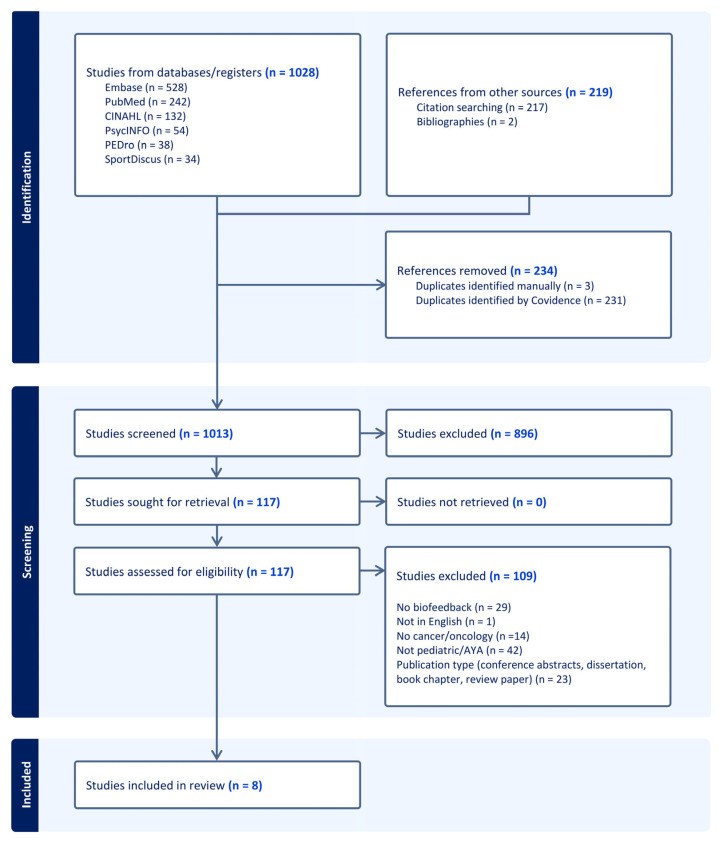
Prisma flow diagram.

Database searches initially identified 1028 articles, with an additional 219 identified via citation searching and bibliographies. After duplicates were removed, the titles of 1013 articles were reviewed for eligibility by two independent reviewers. Following the title review, it was determined that 117 unique article abstracts would be reviewed. A total of 117 articles were retained for the full-text review. A total of 109 (89.2%) full-text articles were excluded for the following reasons: no biofeedback (*n* = 29), not in English (*n* = 1), no cancer/oncology (*n* = 14), not pediatric/AYA (*n* = 42), and publication type (*n* = 23) (Figure 1).

**Figure 2:**
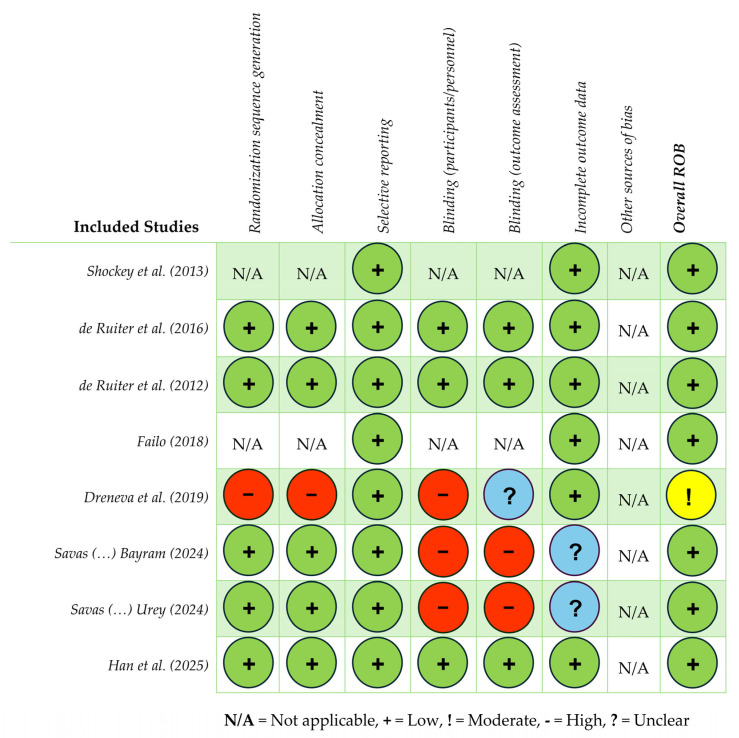
Cochrane Risk of Bias (ROB) Appraisal Tool—7 domains [64,65,66,67,68,69,70,71].

A summary of study characteristics for the eight articles is presented in Table 1. Studies were published between 2012 and 2025 and represent an international dataset from the United States (*n* = 1), China (*n* = 1), the Netherlands (*n* = 2), Italy (*n* = 1), Russia (*n* = 1), and Turkey (*n* = 2). Study designs included the following: double-blind placebo-controlled studies (*n* = 2), randomized placebo-controlled studies (*n* = 2), a prospective quasi-randomized controlled trial (*n* = 1), non-randomized feasibility studies (*n* = 2), and a single-case study (*n* = 1). The methodological quality of included studies was assessed using the Cochrane ROB Tool, modified for non-randomized studies (see Table 1; Figure 2). Overall, the ROB was low for most studies (*n* = 7), with one study assessed as moderate.

**Table 1:** Summary of included articles (N = 8).

Authors (Year), Country	Study Design	Eligibility Criteria	Sample Characteristics	Aims	Biofeedback (BF) Modalities and Measurements	Results	Limitations	Clinical Implications/ Future Research
de Ruiter et al. (2016) [65] The Netherlands	Double-blind parallel placebo-controlled design. Groups: (1) NF, (2) PF. Time Points: (T0) Pre-training, (T1) post-training, (T2) 6 mos post-training.	Patients 8–18 yrs old, treated for brain tumor > 2 yrs prior. Experiencing caregiver-reported neurocognitive complaints.	N = 82 (M age = 13.9 yrs, SD = 3.2; 49% male). Groups: • NF: *n* = 40. • PF: *n* = 40. • Refused qEEG: *n* = 2. • Completed training and included in analysis: *n* = 71.	(1) Assess the efficacy of NF in improving cognitive function among PBTSs. (2) Evaluate the impact of NF on neurocognitive outcomes (e.g., attention, memory, and executive functioning). (3) Compare outcomes of NF group with PF group to determine the specific effects of NF. (4) Provide evidence-based recommendations for the use of NF among PBTSs.	NF EEG	There were no specific treatment effects of NF on neurocognitive functioning. Both groups: PBTSs improved over time on the majority of primary and some secondary outcome measures, with small-to-medium effects. NF does not have favorable effects on neurocognitive and psychosocial functioning in PBTSs as compared with PF.	Participants selected based on parent-reported concerns, not objective measures.	**Implications:** Healthcare providers may need to focus on individualized care plans for PBTSs, given the lack of NF effectiveness in the study. **Research:** Explore alternate, more effective interventions or therapies for PBTSs for improving specific outcomes. Develop and test interventions to improve neurocognitive functioning in PBTSs that minimize the risk of medication side effects, using double-blind, randomized, placebo-controlled trials, including a healthy control group and/or other disease groups. Examine the impact of more targeted BF modalities (e.g., HRV) on PBTS neurocognitive outcomes.
De Ruiter et al. (2012) [66] The Netherlands	Randomized placebo-controlled double-blind trial. Groups: (1a) Experimental group, receiving NF, (1b) placebo group, receiving placebo training, (2) control group (healthy siblings). Time Points: (T0) Pre-training, (T1) post-training, (T2) 6 mos post-training.	Patients 8–18 yrs old, treated for brain tumor >2 yrs prior. Experiencing caregiver-reported neurocognitive complaints. Control: Siblings, 8–18 yrs old.	Total sample: N = 70. Groups: • NF group: *n* = 35. • Placebo group: *n* = 35. • Matched control group: *n* = 35.	(1) Describe the protocol of the PRISMA study, an RCT to investigate the efficacy of NF to improve neurocognitive functioning among PBTSs.	NF qEEG Assessments: Neurocognitive testing, patient/caregiver/teacher reports, qEEG, portable Brainquiry PET NF device.	The study reports the PRISMA intervention methods (study design, participants, intervention, randomization) and procedure (randomization, assessment, power calculation, statistical analyses).	Small sample size. Heterogeneous brain tumor patients (tumor diagnosis, tumor location, age at diagnosis, treatment, time since diagnosis, time since treatment ended). 5 yr survival rates of approximately 65%, some patients may relapse or discontinue participation.	**Implications**: If proven effective, NF could be a valuable addition to current interventions for PBTSs to address common deficits. Automatic threshold adjustment makes NF more feasible in clinical settings and training without active brain monitoring. **Research**: Future studies should test NF feasibility in home/school settings and investigate automated systems further. Explore ways to integrate automated-threshold NF protocols in routine clinical or school-based interventions, and compare outcomes with traditional monitoring protocols.
Dreneva et al. (2020) [68] Russia	Prospective quasi-RCT. Groups: (1) Intervention (BF training and usual activities), (2) waitlist (usual activities). Time Points: (1) Baseline: Days 1–3 after arrival, pre-training. (2) Wk 2: Group A post training, Group B midpoint. (3) Wk 4: Group A 2 wk follow-up, Group B post-training.	Patients <18 yrs old; survivors of PFT and healthy controls (siblings).	Total sample: N = 60. Groups: Intervention: *n* = 35 (M age = 11.4 yrs, SD = 3.5; 23 female). Completed intervention: *n* = 28. Control: *n* = 25 (M age = 12.6 yrs, SD = 2.1; 14 female).	(1) Quantify the postural control state in PFT survivors. (2) Compare PFT survivor stabilometric measurements (postural balance) with healthy controls. (3) Evaluate BF training effects on postural balance enhancement.	Intervention group: BF 6-session training program; used visual feedback about their center-of-pressure location. Stabilometric test measures: Ellipse area, Ellipse Square, mean velocity of CoP, mean root square oscillations in frontal and sagittal planes.	Intervention group: Some change was observed in stability parameters in the 2nd test compared to baseline. Ellipse Square parameter results: there was a substantial decrease in the open-eye condition, indicating improvement in postural stability (positive medium-sized effect). Mean root square oscillation results: this parameter decreased dramatically in the frontal and sagittal planes post-training (medium-sized effects). Both open- and closed-eye conditions were similar. Control group: No significant positive change was observed with open and closed eyes.	Not pure control group. Small sample size. Large age variation in participants. Short duration of training program. Limited and small recruitment pool from one rehabilitation center. Potential influence of disease and treatment on postural impairments, making it difficult to isolate specific contributing factors to balance.	**Implications**: Short-term (2 wk) rehab is insufficient for balance issues in PFT survivors; extended programs (8–12 wks) with BF can enhance postural control strategies. Visual sensory system interference must be considered in therapy plans for balance. Apply the stabilometric method in clinical practice for both diagnostics and rehabilitation to track postural improvements; explore its use for monitoring subtle changes in stability over time. **Research**: Refine and validate the stabilometric method, explore proprioceptive and visual sensory system roles in balance, and ensure reliability and utility in clinical diagnostics and rehabilitation. Validate and refine the BF rehabilitation program for broader application and assess longitudinal outcomes.
Shockey et al. (2013) [64] U.S.A.	Feasibility study. 1-group, non-randomized, repeat-measures design. Time Points: (Sessions 1–4): Pre/post-assessment at each session (60 min).	Children and AYAs undergoing active cancer treatment.	Total sample: N = 12 (M age = 11.0 yrs; ages 8–14; 42% female). Treatment status: • Recently diagnosed: 75%. • Relapsed: 25%.	(1) Assess the feasibility and potential benefits of a 4-session combined relaxation and BF intervention that aims to alleviate procedural distress and increase self-regulation during medical procedures.	HRV via HeartMath emWave System (30 min) for computer with finger sensor in 3/4 sessions; baseline HR and RR in 4/4 sessions. HRV measured pre/post each intervention session using HeartMath emWave System (during sessions 2–4); pre/post-intervention RR and HR measurements for all 4 sessions.	Responses to the parameters and requirements of the study were positive, reported to be well received. State anxiety scores evidenced decreased state anxiety across all sessions, specifically when comparing Session 1 to Session 4. There was improvement in coherence (HRV) scores to a significant degree in Sessions 3- 4. The combination of belly breathing and BF techniques allowed participants to feel in charge of their bodies before their procedures (81% of participants).	Small sample size. No control group. Investigator present at each session providing extra attention to both child and parent during interaction. Reporting discrepancy identified between FACES scale modification and pre-intervention coherence scores. Poor follow-through with homework.	**Implications**: A combined BF and relaxation protocol is feasible for integration into pediatric oncology care and may reduce pre-procedural distress and enhance coping skills and emotional regulation. **Research**: More robust evidence is needed to support the protocol’s efficacy and generalizability, including comparing to a control group and assessing the dose and timing of sessions. Replicate with a control group, expand the sample size, lengthen the intervention (6 vs. 4 sessions), and include follow-up sessions to assess maintenance through coherence measurements. Test with other pediatric illness-specific populations (e.g., sickle cell disease), or add caregiver sessions to increase the utilization of tools and homework engagement.
Failo et al. (2018) [67] Italy	Single-case report. Time Points: (Sessions 1–4): Baseline/post. (Sessions 1, 4): Interviews.	Adolescent patient, undergoing active cancer treatment.	N = 1. Adolescent male diagnosed with ALL.	(1) Evaluate and illustrate a BF protocol (utilizing BF-Assisted Relaxation Training (BART)) integrated into multidisciplinary care, in a format that is acceptable and within a specific treatment time frame. (2) Session Aims: Increase awareness and control of physiological self-regulation to improve pain-related anxiety during oncological invasive procedures and treatments.	Combining relaxation training and practice (deep breathing and mini-PMR) with psychoeducation about pain mechanisms. Modalities: Abdominal (diaphragmatic) breathing exercises, resonance frequency HR and breathing synchronization, breathing app, sEMG training, PMR, HRV, sEMG. BF device: ProComp5 Infiniti System w/Bio Graph Infiniti Software-T7525 (no software version number provided).	The study suggests that BF training may improve emotional regulation due to one’s ability to manage and adapt physiological arousal in response to situational demands during oncological treatment. Outcomes: significant differences were observed between sEMG and HRV measures; RSA was increased; there was an overall increased balance between sympathetic and parasympathetic systems; and there was an increase in awareness about muscle tension and the ability to stimulate blood flow. These results could effectively reflect a decrease in negative psychological symptoms related to pain, suggesting an ability to reframe personal pain perception.	Single-case study. Limited generalizability in outcomes and oncology treatment types. Not manualized. Conducted in Italy with potentially different medical system and treatment model than other countries.	**Implications**: BF may enhance emotional regulation and pain reappraisal due to an increased ability to manage and adapt physiological arousal in response to situational demands during treatment. Knowledge gained from even one BF session can be used to reduce negative physiological arousal. Caregivers may also benefit from increased physiological awareness to support children during treatment (e.g., through procedures and physical symptoms including pain, anxiety). **Research**: Evaluate short-term BF impacts on arousal and pain perception; assess transferability to caregivers. Manualize protocol for greater generalizability and dissemination. Assess the efficacy of BF tools in anxiety- and pain-inducing oncology procedures. Educate providers on session modules and exercise, and the use of targeted BF techniques (e.g., breathing, specific muscle-relaxation, HRV tools), oncology-specific situations (e.g., procedures, needles, scans), and physical symptoms (e.g., pain, anxiety). Conduct additional assessments of the outcomes of simple, accessible, and engaging BF tools as a guide and/or coach in the setting of pediatric/AYA anxiety-provoking and painful medical procedures. Utilize a mixed-methods approach to assessing the reconceptualization of anxiety and pain perception after BF utilization and education.
Hanzade Savas et al. (2024) [70] Turkey	Feasibility study. 1 group, non-randomized. Time Points: (1) Pre-procedure (1 h pre). (2) Procedure: During procedure, reported pain level. (3) Post-procedure (1 h post).	Patients 6–12 yrs old, treated with chemotherapy. No prior experience with respiratory BF in VR.	Total sample: N = 15 pediatric oncology patients and their parents. Intervention: • M age = 9.66 yrs (SD = 2.10). • Gender: 53.3% male. • M duration of port catheter usage: 4.25 mos (SD = 1.81).	(1) Evaluate the feasibility, safety, acceptability, and preliminary effectiveness of a respiratory-based BF VR game, BioVirtualPed, in reducing procedural pain, anxiety, and fear in pediatric oncology patients undergoing port catheter insertions.	Using VR headset to engage in game that guided slow, deep, diaphragmatic breathing. Aimed to promote emotional regulation and physiological calming during medical procedure. Oculus Quest 2 headset; respiratory data captured using ADXL354 accelerometer. Data integrated with Arduino IDE software (no software version number provided).	The intervention group showed significantly lower post-procedure pain, fear, and anxiety scores compared to the control group (*p* < 0.001). There were no significant differences in pre-procedure scores (*p* > 0.05). Mothers reported lower pain and fear levels for their children with higher satisfaction scores (*p* < 0.001). The intervention group exhibited a significantly lower mean RR during procedures (*p* < 0.001). Agreement between child and mother ratings on pain, fear, and anxiety was strong across both groups (*p* < 0.001).	Small sample size and single-site study. No long-term follow-up to assess durability of effects. Lack of physiological measurements beyond RR. Limited diversity in diagnosis and procedure types. Game currently compatible only with specific computer systems and Oculus VR glasses, which are expensive and limit accessibility. Absence of mobile-compatible version restricts daily use and continuous engagement, reducing potential therapeutic impact.	**Implications:** BioVirtualPed may reduce procedural pain, anxiety, and fear in pediatric oncology patients by promoting emotional regulation through respiratory BF. Intervention is feasible and acceptable for use during needle-related procedures and requires minimal provider effort, making it a scalable tool in routine care. **Research:** Evaluate effectiveness across larger and more diverse populations. Explore integration into other pediatric procedures. Assess caregiver involvement to support family-centered care. Conduct RCTs with broader samples, develop and assess mobile-compatible versions for increased accessibility, expand applicability to other medical procedures, and investigate long-term outcomes.
Hanzade Savas et al. (2024) [69] Turkey	Randomized controlled study. Groups: Intervention group wore VR headset and respiratory sensor during needle insertion. Control: Standard of care. Time Points: (1) Pre-procedure (1 h before), (2) during procedure, (3) post-procedure (~3 min after port insertion).	Patients 6–12 yrs old and mothers; with experience inserting port catheter needle ≥1 time.	Total sample: N = 62. Groups: Child group: • Intervention: *n* = 31 (M age = 9.33 yrs, SD = 2.08). • Control: *n* = 31 (M age = 9.74 yrs, SD = 1.76). Mother group: • Intervention: M age = 39.22 yrs (SD = 4.47). • Control group: M age = 39.29 yrs (SD = 3.39).	(1) Does using the BioVirtualPed during port catheter needle insertion reduce the level of procedure-related pain, anxiety, and fear in children? (2) Does using BioVirtualPed during port catheter needle insertion affect RR during the procedure? (3) Does using the BioVirtualPed during port catheter needle insertion increase procedure-related satisfaction?	RR obtained using ADXL354 sensor during breathing exercises on BioVirtualPed VR game. Sensor features: Low noise density, high sensitivity, programmable digital high- and low-pass filters. In BioVirtualPed, participation in game realized through feedback provided by breathing. ADXL354 sensor used in intervention group to obtain respiratory data that would enable interaction with game. Cardiorespiratory signal from ADXL354 sensor: Mean RR per min during needle insertion.	The intervention group showed lower mean pain scores than the control group (*p* < 0.001). Intervention-group mothers reported significantly lower mean pain scores for their child’s needle insertion compared to the control group. There was no difference in pre-procedure fear and anxiety scores between groups (*p* > 0.05 and *p* > 0.05, respectively). Post-procedure fear and anxiety scores were lower in the intervention group (*p* < 0.001 and *p* < 0.001, respectively). Mothers’ post-procedure fear and anxiety scores were lower in the intervention group with a large effect size (*p* < 0.001, d = 1.714 and *p* < 0.001, d = 1.907, respectively). The intervention-group mean RR was lower (*p* < 0.001) and satisfaction scores were higher (*p* < 0.001) during needle insertion. Agreement between children and mothers on pain, fear, and anxiety scores was good and excellent across groups (*p* < 0.001).	No blinding of participant and researcher. Interaction between patients themselves between groups not controllable. Participants with varied experiences (e.g., number of prior needle insertions, healthcare professional performing insertion).	**Implications**: BioVirtualPed showed promising results. Respiratory BF using a VR game format may be feasible and effective and shows potential for reducing pain, anxiety, and fear during procedures in pediatric patients, while improving physiological outcomes by regulating respiration (respiratory rate). It also improves caregiver satisfaction, making it a holistic tool for procedural support. **Research**: Further validation is needed across treatment centers to determine generalizability. Explore the mechanisms underlying the emotional and physiological benefits of the VR-based respiratory feedback system. Assess feasibility and outcomes across diverse treatment settings. Expand dissemination and provider education on the use of respiratory BF for other oncology-related procedures.
Han et al. (2025) [71] China	Two-group, randomized controlled study. Groups: Both groups: Received health education. Intervention: Received 4-unit BF intervention. Time points: (T0) Baseline, (T1) post-intervention, (T2) 4 wk follow-up.	Children: Aged 6–18 yrs, diagnosed with ALL, currently receiving chemotherapy, completed ≥1 invasive procedures per chemotherapy cycle. Able to communicate and express their emotions. Caregiver: ≥18 yrs old, primary caregiver, access to mobile phone/internet, possessing basic reading and writing skills.	Total sample: N = 80 child–caregiver dyads (40 per group). Groups: Intervention (*n* = 40): • M age children = 9.20 yrs (SD = 3.04). • M age caregivers = 38.63 yrs (SD = 8.60). Control (*n* = 40): • M age children = 9.25 yrs (SD = 3.25). • M age caregivers = 39.08 yrs (SD = 6.17).	(1) Assess the effectiveness of a structured BF intervention in alleviating pain, fear, depression, and anxiety related to invasive procedures in children and caregivers with ALL. (2) Improve sleep quality in both children and caregivers.	Intelligent Body and Mind training system 4.0, which integrates HR, respiration, and galvanic skin response. Intervention: 4 structured units: music relaxation, deep breathing training, imaginative relaxation, computer game activity.	The intervention group reported significantly lower post-procedure pain, fear, and worry scores compared to the control group (*p* < 0.001). There were no significant differences in pre-procedure pain, fear, or worry between groups (*p* > 0.05). Caregivers in the intervention showed significantly lower post-intervention anxiety and improved sleep quality compared to controls (*p* < 0.05). No significant differences were found in caregiver depression scores or children’s sleep quality between groups (*p* > 0.05).	Small sample size, limited generalizability due to majority of caregivers being parents, exclusion of younger children (0–6 yrs) and other leukemia types.	**Implications:** BF provides a non-pharmacological approach to managing pain, fear, and negative emotions triggered by invasive procedures in pediatric oncology. Incorporating BF into routine care can improve children’s procedural compliance and emotional regulation. For caregivers, reduced anxiety and improved sleep quality enhance overall well-being and caregiving ability, indirectly benefiting the child’s experience and recovery. **Research:** Explore the optimization of the intervention, including ideal session frequency and duration, and assess long-term outcomes in both children and caregivers. Broader studies could also evaluate generalizability across pediatric populations (e.g., different diseases) and settings. Conduct larger, multicenter RCTs with longer-term follow-up. Use objective sleep and emotional regulation measures to enhance the validity of findings.

Participant ages across the included studies ranged from 6 to 18 years. The oldest sample had a mean age of 13.9 years (SD = 3.2), while the youngest sample included children with mean ages around 9.2 years (SDs ~2–3). One study included a single adolescent participant. Caregiver participants, included in three studies but two distinct datasets, had mean ages of 38.6 and 39.2. All samples were individuals either undergoing active treatment or demonstrating recent survivorship.

Shockey et al. (2013) [64] in the United States present a feasibility study with children aged 8–13 (*n* = 12) undergoing active cancer treatment. The study assessed the feasibility and potential benefits of a four-session combined relaxation and biofeedback intervention to alleviate procedural distress and increase self-regulation during medical procedures. The biofeedback modalities used included the following: (1) HRV, measured pre and post three of four intervention sessions using the HeartMath emWave System during the second, third, and fourth meetings, and (2) pre- and post-intervention respiratory rate and HR measurements for all four intervention sessions. Conclusion: The combined biofeedback and relaxation intervention integrated into routine pediatric oncology care shows promise in reducing pre-procedural distress while supporting emotional regulation and coping.

de Ruiter et al. (2012, 2016) [65,66] in the Netherlands present two studies of a randomized controlled double-blind trial, assessing the efficacy of neurofeedback in improving cognitive function among pediatric brain tumor survivors (PBTSs). The 2012 paper outlines the Pediatric Research on Improving Speed, Memory, and Attention (PRISMA) trial, a double-blind placebo-controlled study comparing 30 sessions of EEG-based neurofeedback versus placebo feedback (PF) in PBTSs aged 8–18 (*n* = 70), with a sibling control group. The 2016 paper reports outcomes from an expanded sample (*n* = 82), showing no significant differences between neurofeedback and PF groups in cognitive functions like attention, memory, or executive functioning. Both groups improved similarly over time, suggesting non-specific treatment effects. Conclusion: Despite neurofeedback being a specific type of biofeedback that targets the central nervous system, it did not show unique benefits over the placebo. Improvements may have been due to general engagement or expectancy effects rather than the modality itself, underscoring the need for more-targeted and evidence-based interventions for PBTSs.

Failo et al. (2018) [67] in Italy present a single-case report assessing a brief four-session biofeedback protocol utilizing Biofeedback-Assisted Relaxation Training (BART), integrated into multidisciplinary care. Delivered to a male adolescent patient with acute lymphocytic leukemia (ALL), sessions aimed to increase awareness and control of physiological processes. The assessment and training of self-control of physiological functions was based on a pre–post session-by-session evaluation. Sessions included relaxation training (deep breathing and mini-progressive muscle relaxation (PMR)) alongside psychoeducation on pain mechanisms and the role of distress in amplifying pain and inflammation. Biofeedback modalities during the four sessions included the following: abdominal (diaphragmatic) breathing exercises, resonance frequency (HR and breathing synchronization) breathing with an app-based pacing guide for inhalation and exhalation, sEMG training, and PMR. Conclusion: Biofeedback training may improve emotional regulation by helping manage physiological arousal and reconceptualize anxiety. Biofeedback also promotes caregiver involvement by teaching strategies to recognize and respond to mental distress during medical procedures.

Dreneva and Skvortsov (2020) [68] in Russia conducted a prospective quasi-randomized controlled trial evaluating biofeedback training effects on postural balance enhancement among survivors of posterior fossa tumors (PFTs) and healthy controls. Using stabilometric measures, the study compared PFT survivors (*n* = 35) with healthy controls (*n* = 25) and assessed changes over a four-week period with staggered intervention delivery. The study found moderate improvements in sway and oscillation metrics after a short, game-based biofeedback intervention in children recovering from cerebellar tumors. Conclusion: Integrating biofeedback into comprehensive neuro-oncology rehabilitation may help improve functional outcomes for this vulnerable population. While some findings suggest biofeedback benefits for postural control, the study had significant limitations.

Savas et al. (2024) [69,70] in Turkey present two feasibility studies evaluating BioVirtualPed, a virtual reality (VR)-based respiratory biofeedback game developed to reduce procedural distress among pediatric oncology patients undergoing port needle insertions. The first study (*n* = 15, M age 9.66) assessed the usability and safety of an Oculus Quest 2-based intervention, which guides slow diaphragmatic breathing using an integrated biosensor and Arduino software (no software version number provided). A second, larger study (*n* = 62) found that BioVirtualPed significantly reduced children’s post-procedure pain, fear, and anxiety, improved respiratory regulation, and enhanced caregiver satisfaction. Conclusion: BioVirtualPed demonstrates feasibility and clinical potential in managing procedural distress via respiratory feedback; however, its broader use is currently limited by device compatibility and cost.

Han et al. (2025) [71] in China conducted a randomized controlled study with 80 child–caregiver dyads, including children aged 6–18 undergoing chemotherapy for Acute Lymphoblastic Leukemia. The study evaluated the effects of a structured four-session biofeedback intervention using the Intelligent Body and Mind training system 4.0 on procedural pain, fear, worry, and caregiver distress. Biofeedback modalities included HR, respiration, and galvanic skin response. Conclusion: The structured biofeedback intervention shows significant promise in reducing post-procedure pain, fear, and worry in children and improving caregiver anxiety and sleep quality.

Examining these studies together, this systematic review identified only 8 studies meeting the inclusion criteria out of the 1013 screened, underscoring the scarcity of biofeedback research in pediatric and AYA oncology. Types of Cancer Studied: The included studies focused on diverse cancer types. Several investigated brain tumor survivors [65,66], while others included children and adolescents with leukemia, lymphoma, or mixed oncology diagnoses [64,71]. Three of the studies targeted survivors of central nervous system malignancies and the associated neurocognitive deficits [65,66,68], while the rest addressed symptom management in those undergoing active cancer treatment [64,67,70,71]. Types of Biofeedback Used: Neurofeedback (EEG biofeedback) was used in studies aiming to improve cognitive functioning in pediatric brain tumor survivors [65,66]. HRV, respiratory biofeedback, and GSR were used to modulate autonomic function, aiming to reduce anxiety and improve stress regulation [70]. Stabilometric biofeedback aimed to improve posture and balance [68]. Some interventions combined biofeedback with complementary techniques, such as PMR and guided imagery, reflecting an integrative approach to symptom management [64,67]. Study Designs and Methodological Quality: Included studies comprised randomized placebo-controlled trials, double-blind designs, quasi-experimental studies, feasibility studies, and one single-case report. While most studies demonstrated a low risk of bias, sample sizes were small (ranging from a single case to 82 participants), and heterogeneity in study designs limited generalizability. Key Outcomes Evaluated: (1) Neurocognitive functioning (attention, memory, executive function) in brain tumor survivors, with neurofeedback showing no specific treatment effects compared to the placebo [65,66], while posture- and balance-focused biofeedback in brain tumor survivors showed positive effects [68]; (2) anxiety, stress, and emotional regulation, with HRV, respiratory, and GSR biofeedback demonstrating positive effects toward reduced anxiety and improved autonomic balance [64,70,71]; (3) the feasibility and acceptability of biofeedback interventions in pediatric oncology settings, generally demonstrating that interventions were well tolerated and feasible to implement [64,70].

Notably, neurofeedback was the only modality that did not demonstrate positive effects beyond the placebo. Neurofeedback, which targets the central nervous system, differs from the other studied modalities that primarily target the peripheral nervous system with an emphasis on autonomic regulation. While a single study is insufficient to conclude that neurofeedback is ineffective, it remains the most logistically complex, time-consuming, and expensive of the biofeedback modalities. In contrast, interventions targeting the autonomic nervous system, such as HRV biofeedback, are more feasible to implement and demonstrated promising outcomes in the included studies, suggesting that focusing future research and clinical application on autonomic-focused biofeedback modalities may be the most efficient and impactful path forward.

## 4. Discussion

Cancer is a significant global health issue for this age group, with approximately 400,000 children [72,73] and 1.3 million AYAs being diagnosed annually [74]. Although survival rates have improved, cancer treatment in children, adolescents, and young adults often disrupts normal development and involves repeated invasive procedures, prolonged hospitalizations, and ongoing uncertainty. These factors can lead to heightened anxiety, sadness, and procedural distress, along with a range of short-term side effects (such as pain, headaches, fatigue, and self-regulation challenges) and longer-term treatment-related effects (such as emotional distress, cognition, posture/balance) [75,76,77]. As such, continuing to identify and develop effective and multidisciplinary interventions is essential. Biofeedback is a short-term, skills-based intervention that provides a patient-centered, caregiver-centered, or provider-centered approach. Despite demonstrated efficacy in various physiological and psychological conditions among broader pediatric and adult studies, data on the use of biofeedback specifically in pediatric and AYA oncology remains limited and underrepresented. In our review, this gap is highlighted, as fewer than 1% of the initial articles in our search met our age criteria. Additionally, while the inclusion criteria included individuals aged 0–39, these studies represent only those under 18 years old. This represents a significant opportunity for future investigation, particularly since AYAs represent approximately six times the number of cancers diagnosed in children aged 0–14 [78]. As digital natives with familiarity and comfort with technology, wearable devices, apps, and VR platforms, AYAs may be uniquely positioned to benefit from and participate in biofeedback interventions and self-monitoring “homework” assignments, within and between sessions.

As we routinely provide medications for treatment-related side effects and offer a range of adjuvant therapies as part of oncological treatment, it is both logical and necessary to consider biofeedback and relaxation training, along with their associated self-monitoring tools, as feasible, relevant, and effective components of comprehensive oncology care. These interventions align with a patient-centered model of care and empower children and AYAs (and caregivers) with tangible skills to regulate their individual physiological and emotional responses to treatment. The wide variability in cancer types and treatment trajectories among children and AYAs largely renders a “one size fits all” standard of care insufficient in meeting the diverse needs of this age and illness population.

A 2020 meta-analysis [6] concluded that biofeedback in pediatric populations is effective with large effect sizes, despite the variability in the quality of available research. Included in the meta-analysis were studies focused on asthma, anxiety, pediatric headaches, chronic pain, cystic fibrosis, cerebral palsy, and dyslexia, with headaches being the most studied condition. Another meta-analysis examined biofeedback efficacy in pediatric headaches specifically, finding that it reduced migraine frequency, attack duration, and headache intensity compared to a waiting-list control [56].

Given the numerous short- and long-term effects of pediatric/AYA cancer treatment, biofeedback represents a promising, developmentally appropriate intervention. This lack of research and attention in pediatric and AYA oncology may be due to systemic and educational barriers, including the following: staffing and budget constraints for equipment and training, a lack of prioritization, overall training and continuing education gaps, funding limitations for clinical care and research, and methodological and recruitment challenges unique to this population. Future research directions and clinical implications are now presented as a call to action for biofeedback education and implementation for our patients.

### 4.1. Future Research

It will be critical for future research to seek collaboration between biofeedback experts and oncology centers to advance this field and create accessible, effective tools for children and AYAs with cancer. Future studies should consider biofeedback efficacy for different cancer diagnoses and treatment types, since outcomes may vary. For instance, brain tumor patients may respond differently than those with sarcoma (e.g., general autonomic nervous system dysregulation versus specific neurological deficits due to tumor type). Understanding when biofeedback is most effective—whether during medical procedures, for managing ongoing symptoms, or for reducing scan-related anxiety—can help guide more-targeted use, especially given the limited availability of trained providers, time, and equipment [40]. While at least four sessions of biofeedback has been suggested as a minimum “dose” to achieve short-term benefits [63], the optimal number of sessions likely depends on the specific condition and clinical setting. Results across studies have been mixed, and there are currently no consistent guidelines for dosage. Importantly, although four sessions may yield short-term improvements, especially in acute contexts, long-term effects have not been well studied. A greater number of sessions may be necessary to support lasting improvements in autonomic nervous system regulation.

In some cases, even a single session has been associated with noticeable improvements reported by patients [54]. Moreover, data show that HRV biofeedback delivered prior to surgery can lead to immediate benefits in perioperative care and postoperative outcomes, suggesting that even brief interventions may be effective when well-timed [79,80]. Still, more research is needed to determine how session frequency and duration influence sustained clinical outcomes. Future studies should address the gaps presented in this review through well-designed randomized studies with control groups, larger sample sizes, and longitudinal follow-up.

Given the global burden of cancer and mental health among this population, the cultural and contextual fit of biofeedback equipment and applications is significantly understudied. Variability in access to technology, internet connection, digital literacy, cultural beliefs about physiologically focused interventions, and participation preferences should inform how these tools are received and utilized. Future biofeedback development and implementation should be guided by culturally responsive and equitable approaches to support broad relevance and accessibility across diverse pediatric and AYA oncology populations. A mixed-methods design including qualitative feedback from AYAs can be utilized to develop biofeedback tools in different languages and use culturally diverse avatars or imagery in apps and VR platforms.

### 4.2. Clinical Implications

There are practical ways to clinically bring biofeedback into pediatric/AYA oncology care. While obtaining biofeedback certification through the BCIA can present time and cost barriers, any licensed clinician—including psychologists, psychiatrists, social workers, and integrative medicine providers—can pursue both training and certification in biofeedback [81]. Importantly, licensed providers who have completed the initial didactic training may begin using biofeedback with patients under mentorship, allowing them to gain practical experience and serve patients while working toward full certification.

Once a provider within a clinic is BCIA-certified, they can serve as a mentor for others, helping to train and support new clinicians, and gradually expanding access to biofeedback services across the organization. In addition to full certification, individuals with non-licensed degrees can become biofeedback technicians, or receive a focused HRV-only certification.

To further increase clinician knowledge and confidence, clinics can incorporate webinars, in-service trainings, and grand rounds as educational tools. The BCIA and AAPB offer regular webinars and host conferences on various biofeedback-related topics. Introducing biofeedback education early in medical or clinical training, particularly for those rotating through areas like pediatric oncology, can lay a stronger foundation for future use and integration.

When provider training or clinical bandwidth is limited, cardiovascular and respiratory biofeedback—especially HRV biofeedback—emerges as one of the most accessible and practical options, particularly in oncology settings. As a general self-regulation tool, it is often easier to implement across a wide range of patients, including children and AYAs.

Integrating biofeedback with relaxation techniques or virtual reality (VR) shows promising potential to support patients—and even caregivers—through challenging parts of the treatment journey, such as procedural distress [67,82,83]. Given the growing evidence for the feasibility and benefits of VR in pediatrics, many patients may find technology-enhanced, gamified approaches more appealing, especially younger populations or student athletes [84]. In clinical environments where patients frequently experience physical monitoring and a loss of control, biofeedback can also provide a sense of agency—allowing them to engage with their bodies from a place of autonomy and empowerment.

Additionally, mobile biofeedback apps offer clinicians and patients a flexible, cost-effective way to learn and practice biofeedback techniques outside of clinical sessions. Many of these apps provide built-in guidance, support home practice, and can help reinforce key biofeedback concepts over time—making them accessible with internet access even for clinicians with limited formal biofeedback training. Since apps are continually changing and their validation data is not readily and systematically available, recommendations for use cannot be reported. Given the rapid pace of technological change, clinicians are encouraged to routinely explore new tools and evaluate their quality, usability, and clinical relevance as the landscape continues to evolve.

While biofeedback implementation can be complex, simplifying both the process and equipment is essential for integration into oncology care. The minimal viable infrastructure for HRV biofeedback includes the following: (1) at least one clinician completing 16 h BCIA-accredited introductory HRV biofeedback training; (2) the selection of a single mobile application paired with an affordable HRV sensor that can be used in clinic sessions and shared with patients for home practice; and (3) a 5-day baseline HRV assessment followed by 6–10 weeks of daily HRV biofeedback practice for 10–20 min per day, with continued HRV tracking using standardized HRV measures, as outlined by Shaffer and Ginsberg, 2017 [85]. Future trials should evaluate the feasibility, effectiveness, and intervention adherence of these streamlined protocols in time-constrained oncology settings.

### 4.3. Limitations

The included studies exhibited small sample sizes and significant heterogeneity in intervention protocols (e.g., duration, biofeedback modality), outcome measures, and sample characteristics, limiting comparability and raising concerns about efficacy. Study quality and methodological rigor (e.g., small sample sizes, lack of control group and longitudinal assessment) further limit the interpretation and strength of results. The exclusion of non-English-language publications may have restricted this review’s scope, and publication bias may be present since studies with null or negative findings are published less often. The included studies presented heterogeneous patient groups and variability in intervention durations (e.g., the Dreneva et al. study [68] provided only two sessions), raising questions about efficacy. The ROB across studies further challenges the generalizability of results and undermines methodological rigor.

## 5. Conclusions

Oncology care for this unique age group demands flexible, innovative, and individualized treatments due to the complexity and variability in diagnoses and treatments. Biofeedback shows potential as an adjunctive, integrative intervention to enhance self-regulation, reduce distress, and improve quality of life throughout the cancer trajectory. Rigorous research and clinical direction are needed to support its application and specificity in cancer care, and to understand its long-term benefits and potential limitations in pediatric and AYA oncology settings.

## Data Availability

The original contributions presented in this study are included in the article/Supplementary Materials. Further inquiries can be directed to the corresponding author.

## Supplementary Materials

The following supporting information can be downloaded at https://www.mdpi.com/article/10.3390/children12080998/s1, Table S1: Database search strategies.

## Abbreviations

The following abbreviations are used in this manuscript:

AYA	Adolescent and young adult
ALL	Acute lymphocytic leukemia
AAPB	Association for Applied Psychophysiology and Biofeedback
BF	Biofeedback
BCIA	Biofeedback Certification International Alliance
EEG	Electroencephalography
EMG	Electromyography
HR	Heart rate
HRV	Heart rate variability
ISNR	International Society for Neurofeedback and Research
mos	month
NF	Neurofeedback
PBTS	Pediatric brain tumor survivor
PF	Placebo feedback
PFT	Posterior fossa tumor
PMR	Progressive muscle relaxation
PTSD	Post-traumatic stress disorder
ROB	Risk of bias
sEMG	Surface electromyography
VR	Virtual reality
wk	week
yr	year
