# Supplementary material for: Biofeedback in Pediatric, Adolescent, and Young Adult Cancer Care: A Systematic Review
Source: Children (Basel). 2025 Jul 29;12(8):998. doi: 10.3390/children12080998 (PMC12384105; doi:10.3390/children12080998)
Supplement: Supplementary file 1 [file children-12-00998-s001.zip › children-3714783 Supplemental Materials.pdf]

Table S1. Database Search Strategies

#1	Search strategy components – PubMed/MEDLINE (NLM)
**A**	Biofeedback
	("Biofeedback, Psychology"[Mesh] OR "Feedback, Sensory"[Mesh] OR "Feedback, Psychological"[Mesh] OR "Neurofeedback"[Mesh] OR ((“biofeedback”[TIAB] OR “bio-feedback”[TIAB] OR “neurofeedback”[TIAB]) AND (therap* OR treatment* OR intervention*)))
**B**	Cancer/Childhood Cancer
	(cancer OR cancers OR cancerous OR oncology OR oncolog* OR neoplasm OR neoplasms OR neoplasm* OR carcinoma OR carcinom* OR tumor OR tumour OR tumors OR tumours OR malignan* OR malignant OR "hematooncological" OR "hemato oncological" OR "hemato-oncological" OR “hematologic neoplasms” OR hematolo*) OR ("bone marrow transplant*" OR leukemi* OR leukaemi* OR lymphom* OR hodgkin* OR "T-cell" OR "B-cell" OR "non-hodgkin" OR sarcom* OR Ewing* OR osteosarcom* OR wilms* OR nephroblastom* OR neuroblastom* OR rhabdomyosarcom* OR teratom* OR hepatom* OR hepatoblastom* OR medulloblastom* OR PNET* OR "neuroectodermal tumors, primitive" OR retinoblastom* OR meningiom* OR gliom*)
**C**	Pediatric, AYA
	((Infan* OR newborn* OR “new-born*” OR perinat* OR neonat* OR baby OR baby* OR babies OR toddler* OR “minors”[MH] OR boy OR boys OR boyfriend OR boyhood OR girl* OR kid OR kids OR child OR child* OR children* OR schoolchild* OR schoolchild OR “school child”[tiab] OR “school child*”[tiab] OR adolescen* OR juvenil* OR youth* OR teen* OR “under age*” OR underage* OR pubescen* OR prepubescen* OR pediatrics[mh] OR pediatric* OR paediatric* OR peadiatric* OR school[tiab] OR school*[tiab] OR preschool[tiab] OR preschool*[tiab] OR prematur* OR preterm*) OR (teen*[TIAB] OR youth*[TIAB] OR adolescen*[TIAB] OR juvenile*[TIAB] OR “young adult*”[TIAB] OR “young person*”[TIAB] OR “young individual*”[TIAB] OR “young people*”[TIAB] OR “young population*”[TIAB] OR “young man”[TIAB] OR “young men”[TIAB] OR “young woman”[TIAB] OR “young women”[TIAB] OR youngster*[TIAB] OR “first-grader*”[TIAB] OR “second-grader*”[TIAB] OR “third-grader*”[TIAB] OR “fourth-grader*”[TIAB] OR “fifth-grader*”[TIAB] OR “sixth-grader*”[TIAB] OR “seventh-grader*”[TIAB] OR highschool* OR college* OR “secondary school*”[TIAB] OR “secondary education*”[TIAB] OR “high school*”[TIAB] OR “high education”[TIAB] OR

	adolescent[MH] OR “young adult”[MH]))
**D**	Limit to: English, Human
	(English[lang] NOT (animals[mh] NOT humans [mh]))
**E**	A AND B AND C AND D

#2	**Search strategy components – EMBASE (Elsevier)**
**A**	Biofeedback
	('biofeedback'/exp OR 'sensory feedback'/exp OR 'psychological feedback'/exp OR 'neurofeedback'/exp OR ((biofeedback:ti,ab OR 'bio feedback':ti,ab OR neurofeedback:ti,ab) AND (therap* OR treatment* OR intervention*)))
**B**	Cancer/Childhood Cancer
	('cancer'/exp OR cancer OR 'cancers'/exp OR cancers OR cancerous OR 'oncology'/exp OR oncology OR oncolog* OR 'neoplasm'/exp OR neoplasm OR 'neoplasms'/exp OR neoplasms OR neoplasm* OR 'carcinoma'/exp OR carcinoma OR carcinom* OR 'tumor'/exp OR tumor OR 'tumour'/exp OR tumour OR tumors OR tumours OR malignan* OR malignant OR 'hematooncological' OR 'hemato oncological' OR 'hemato-oncological' OR 'hematologic neoplasms' OR hematolo*) OR ("bone marrow transplant*" OR leukemi* OR leukaemi* OR lymphom* OR hodgkin* OR "T-cell" OR "B-cell" OR "non-hodgkin" OR sarcom* OR Ewing* OR osteosarcom* OR wilms* OR nephroblastom* OR neuroblastom* OR rhabdomyosarcom* OR teratom* OR hepatom* OR hepatoblastom* OR medulloblastom* OR PNET* OR "neuroectodermal tumors, primitive" OR retinoblastom* OR meningiom* OR gliom*)
**C**	Pediatric, AYA
	((Infan* OR newborn* OR new-born* OR perinat* OR neonat* OR baby OR baby* OR babies OR toddler* OR 'minor (person)'/exp OR boy OR boys OR boyfriend OR boyhood OR girl* OR kid OR kids OR child OR child* OR children* OR schoolchild* OR schoolchild OR “school child”:ti,ab OR “school child*”:ti,ab OR adolescen* OR juvenil* OR youth* OR teen* OR “under age*” OR underage* OR pubescen* OR prepubescen* OR 'pediatrics'/exp OR pediatric* OR paediatric* OR peadiatric* OR school:ti,ab OR school*:ti,ab OR preschool:ti,ab OR preschool*:ti,ab OR prematur* OR preterm*) OR (teen*:ti,ab OR youth*:ti,ab OR adolescen*:ti,ab OR juvenile*:ti,ab

	OR “young adult*”:ti,ab OR “young person*”:ti,ab OR “young individual*”:ti,ab OR “young people*”:ti,ab OR “young population*”:ti,ab OR “young man”:ti,ab OR “young men”:ti,ab OR “young woman”:ti,ab OR “young women”:ti,ab OR youngster*:ti,ab OR “first-grader*”:ti,ab OR “second-grader*”:ti,ab OR “third-grader*”:ti,ab OR “fourth-grader*”:ti,ab OR “fifth-grader*”:ti,ab OR “sixth-grader*”:ti,ab OR “seventh-grader*”:ti,ab OR highschool* OR college* OR “secondary school*”:ti,ab OR “secondary education*”:ti,ab OR “high school*”:ti,ab OR “high education”:ti,ab OR 'adolescent'/exp OR 'young adult'/exp))
**D**	Limit to: English, Human
	([english]/lim AND 'human'/de)
**E**	A AND B AND C AND D

#3	**Search strategy components – CINAHL (EBSCO)**
**A**	Biofeedback
	((MH "Biofeedback") OR (MH "Feedback") OR (((TI “biofeedback”) OR (TI “bio-feedback”) OR (TI “neurofeedback”) OR (AB “biofeedback”) OR (AB “bio-feedback”) OR (AB “neurofeedback”)) AND (<br>* OR treatment* OR intervention*)))
**B**	Cancer/Childhood Cancer
	(cancer OR cancers OR cancerous OR oncology OR oncolog* OR neoplasm OR neoplasms OR neoplasm* OR carcinoma OR carcinom* OR tumor OR tumour OR tumors OR tumours OR malignan* OR malignant OR "hematooncological" OR "hemato oncological" OR "hemato-oncological" OR “hematologic neoplasms” OR hematolo*) OR ("bone marrow transplant*" OR leukemi* OR leukaemi* OR lymphom* OR hodgkin* OR "T-cell" OR "B-cell" OR "non-hodgkin" OR sarcom* OR Ewing* OR osteosarcom* OR wilms* OR nephroblastom* OR neuroblastom* OR rhabdomyosarcom* OR teratom* OR hepatom* OR hepatoblastom* OR medulloblastom* OR PNET* OR "neuroectodermal tumors, primitive" OR retinoblastom* OR meningiom* OR gliom*)
**C**	Pediatric, AYA
	((Infan* OR newborn* OR “new-born*” OR perinat* OR neonat* OR baby OR baby* OR babies OR toddler* OR (MH “Minors (Legal)”) OR boy OR boys OR boyfriend OR boyhood OR girl* OR kid OR kids OR (MH "Child+") OR child OR child* OR

	children* OR schoolchild* OR schoolchild OR (TI “school child*”) OR (AB “school child*”) OR adolescen* OR juvenil* OR youth* OR teen* OR “under age*” OR underage* OR pubescen* OR prepubescen* OR (MH "Pediatrics+") OR pediatric* OR paediatric* OR peadiatric* OR (TI “school*”) OR (AB “school*”) OR (TI “preschool*”) OR (AB “preschool*”) OR prematur* OR preterm*) OR ((TI “teen*”) OR (AB “teen*”) OR (TI “youth*”) OR (AB “youth*”) OR (TI “adolescen*”) OR (AB “adolescen*”) OR (TI “juvenile*”) OR (AB “juvenile*”) OR (TI “young adult*”) OR (AB “young adult*”) OR (TI “young person*”) OR (AB “young person*”) OR (TI “young individual*”) OR (AB “young individual*”) OR (TI “young people*”) OR (AB “young people*”) OR (TI “young population*”) OR (AB “young population*”) OR (TI “young man”) OR (AB “young man”) OR (TI “young men”) OR (AB “young men”) OR (TI “young woman”) OR (AB “young woman”) OR (TI “young women”) OR (AB “young women”) OR (TI “youngster*”) OR (AB “youngster*”) OR (TI “first-grader*”) OR (AB “first-grader*”) OR (TI “second-grader*”) OR (AB “second-grader*”) OR (TI “third-grader*”) OR (AB “third-grader*”) OR (TI “fourth-grader*”) OR (AB “fourth-grader*”) OR (TI “fifth-grader*”) OR (AB “fifth-grader*”) OR (TI “sixth-grader*”) OR (AB “sixth-grader*”) OR (TI “seventh-grader*”) OR (AB “seventh-grader*”) OR highschool* OR college* OR (TI “secondary school*”) OR (AB “secondary school*”) OR (TI “secondary education*”) OR (AB “secondary education*”) OR (TI “high school*”) OR (AB “high school*”) OR (TI “high education”) OR (AB “high education”) OR (MH "Adolescence+") OR (MH "Young Adult")))
**D**	Limit to: English, Human
	**Limiters** - English Language; Human
**E**	A AND B AND C AND D

#4	**Search strategy components – SPORTDiscus (EBSCO)**
**A**	Biofeedback
	(((TI “biofeedback”) OR (TI “bio-feedback”) OR (TI “neurofeedback”) OR (AB “biofeedback”) OR (AB “bio-feedback”) OR (AB “neurofeedback”)) AND (therap* OR treatment* OR intervention*)))
**B**	Cancer/Childhood Cancer
	(cancer OR cancers OR cancerous OR oncology OR oncolog* OR neoplasm OR neoplasms OR neoplasm* OR carcinoma OR carcinom* OR tumor OR tumour OR tumors OR tumours OR malignan* OR malignant OR "hematooncological" OR

	"hemato oncological" OR "hemato-oncological" OR “hematologic neoplasms” OR hematolo*) OR ("bone marrow transplant*" OR leukemi* OR leukaemi* OR lymphom* OR hodgkin* OR "T-cell" OR "B-cell" OR "non-hodgkin" OR sarcom* OR Ewing* OR osteosarcom* OR wilms* OR nephroblastom* OR neuroblastom* OR rhabdomyosarcom* OR teratom* OR hepatom* OR hepatoblastom* OR medulloblastom* OR PNET* OR "neuroectodermal tumors, primitive" OR retinoblastom* OR meningiom* OR gliom*)
**C**	Pediatric, AYA
	((Infan* OR newborn* OR “new-born*” OR perinat* OR neonat* OR baby OR baby* OR babies OR toddler* OR “Minors” OR boy OR boys OR boyfriend OR boyhood OR girl* OR kid OR kids OR (DE "CHILDREN") OR child OR child* OR children* OR schoolchild* OR schoolchild OR (TI “school child*”) OR (AB “school child*”) OR adolescen* OR juvenil* OR youth* OR teen* OR “under age*” OR underage* OR pubescen* OR prepubescen* OR (DE "PEDIATRICS") OR pediatric* OR paediatric* OR peadiatric* OR (TI “school*”) OR (AB “school*”) OR (TI “preschool*”) OR (AB “preschool*”) OR prematur* OR preterm*) OR ((TI “teen*”) OR (AB “teen*”) OR (TI “youth*”) OR (AB “youth*”) OR (TI “adolescen*”) OR (AB “adolescen*”) OR (TI “juvenile*”) OR (AB “juvenile*”) OR (TI “young adult*”) OR (AB “young adult*”) OR (TI “young person*”) OR (AB “young person*”) OR (TI “young individual*”) OR (AB “young individual*”) OR (TI “young people*”) OR (AB “young people*”) OR (TI “young population*”) OR (AB “young population*”) OR (TI “young man”) OR (AB “young man”) OR (TI “young men”) OR (AB “young men”) OR (TI “young woman”) OR (AB “young woman”) OR (TI “young women”) OR (AB “young women”) OR (TI “youngster*”) OR (AB “youngster*”) OR (TI “first-grader*”) OR (AB “first-grader*”) OR (TI “second-grader*”) OR (AB “second-grader*”) OR (TI “third-grader*”) OR (AB “third-grader*”) OR (TI “fourth-grader*”) OR (AB “fourth-grader*”) OR (TI “fifth-grader*”) OR (AB “fifth-grader*”) OR (TI “sixth-grader*”) OR (AB “sixth-grader*”) OR (TI “seventh-grader*”) OR (AB “seventh-grader*”) OR highschool* OR college* OR (TI “secondary school*”) OR (AB “secondary school*”) OR (TI “secondary education*”) OR (AB “secondary education*”) OR (TI “high school*”) OR (AB “high school*”) OR (TI “high education”) OR (AB “high education”) OR (DE "TEENAGERS") OR (DE "YOUNG adults")))
**D**	Limit to: English
	**Narrow by Language:** - english
**E**	A AND B AND C AND D

#5	**Search strategy components - PsycINFO (OVID)**

**A**	Biofeedback
	biofeedback/ or feedback/ or neurotherapy/ or ((biofeedback or bio-feedback or neurofeedback).ab,ti. and (therap* or treatment* or intervention*).mp.)
**B**	Cancer/Childhood Cancer
	(cancer or cancers or cancerous or oncology or oncolog* or neoplasm or neoplasms or neoplasm* or carcinoma or carcinom* or tumor or tumour or tumors or tumours or malignan* or malignant or "hematooncological" or "hemato oncological" or "hemato-oncological" or "hematologic neoplasms" or hematolo* or "bone marrow transplant*" or leukemi* or leukaemi* or lymphom* or hodgkin* or "T-cell" or "B-cell" or "non-hodgkin" or sarcom* or Ewing* or osteosarcom* or wilms* or nephroblastom* or neuroblastom* or rhabdomyosarcom* or teratom* or hepatom* or hepatoblastom* or medulloblastom* or PNET* or "neuroectodermal tumors, primitive" or retinoblastom* or meningiom* or gliom*).mp. [mp=title, abstract, heading word, table of contents, key concepts, original title, tests & measures, mesh word]
**C**	Pediatric, AYA
	((Infan* OR newborn* OR "new-born*" OR perinat* OR neonat* OR baby OR baby* OR babies OR toddler* OR "minors" OR boy OR boys OR boyfriend OR boyhood OR girl* OR kid OR kids OR child OR child* OR children* OR schoolchild* OR schoolchild OR (school child).ab,ti. OR (school child*).ab,ti. OR adolescen* OR juvenil* OR youth* OR teen* OR "under age*" OR underage* OR pubescen* OR prepubescen* OR exp Pediatrics/ OR pediatric* OR paediatric* OR peadiatric* OR school.ab,ti. OR school*.ab,ti. OR preschool.ab,ti. OR preschool*.ab,ti. OR prematur* OR preterm*) OR (teen*.ab,ti. OR youth*.ab,ti. OR adolescen*.ab,ti. OR juvenile*.ab,ti. OR (young adult*).ab,ti. OR (young person*).ab,ti. OR (young individual*).ab,ti. OR (young people*).ab,ti. OR (young population*).ab,ti. OR (young man).ab,ti. OR (young men).ab,ti. OR (young woman).ab,ti. OR (young women).ab,ti. OR youngster*.ab,ti. OR (first-grader*).ab,ti. OR (second-grader*).ab,ti. OR (third-grader*).ab,ti. OR (fourth-grader*).ab,ti. OR (fifth-grader*).ab,ti. OR (sixth-grader*).ab,ti. OR (seventh-grader*).ab,ti. OR highschool* OR college* OR (secondary school*).ab,ti. OR (secondary education*).ab,ti. OR (high school*).ab,ti. OR (high education).ab,ti. OR adolescent OR "young adult"))
**D**	Limit to: English, Human
	limit to (human and english language)

**E**	A AND B AND C AND D

**#6**	**Search strategy components – PEDro (NeuRA) https://search.pedro.org.au/advanced-search**
**A**	Biofeedback
	Title/Abstract: Biofeedback
**B**	Cancer
	Subdiscipline: oncology
**C**	A AND B

Abbreviations: Mesh/mh/MH = Subject Heading, ti/TI = Title, ab/AB = Abstract, exp = Explode (includes more specific terms)

Search Filters (age specific) consulted/adapted:

61. Leclercq, E.; Leeflang, M.M.; van Dalen, E.C.; Kremer, L.C. Validation of search filters for identifying pediatric studies in PubMed. *J Pediatr* **2013**, *162*, 629-634 e622, doi:10.1016/j.jpeds.2012.09.012.
62. Tessier, V.; Lacourse, M.; Canadian Health Libraries Association. Adolescents and young adults (Search filter, age specific). Available online: https://extranet.santecom.qc.ca/wiki/!biblio3s/doku.php?id=concepts:adolescents-et-jeunes-adultes (accessed on 6/5/2025).
